# Employment equity groups’ experience of inclusion and commitment to the CAF

**DOI:** 10.3389/fpsyg.2024.1323474

**Published:** 2024-05-15

**Authors:** Jennifer M. Peach, Joelle Laplante, Kayla Boileau

**Affiliations:** ^1^Department of National Defence, Director General Military Personnel Research and Analysis, Ottawa, ON, Canada; ^2^School of Psychology, University of Ottawa, Ottawa, ON, Canada

**Keywords:** inclusion, microaggressions, job satisfaction, affective commitment, Indigenous, persons with disabilities, racialized groups, gender

## Abstract

The Canadian Armed Forces (CAF) are subject to the Employment Equity Act, which requires federally regulated employers to identify and eliminate barriers to the employment of designated groups (women, Indigenous peoples, persons with disabilities (PwD), and racialized members), and establish short-term, numerical goals to address underrepresentation. Addressing employment barriers experienced by these equity seeking groups is one of the CAF’s key priorities. The objective of this study is to examine group differences in feelings of inclusion (i.e., relatedness, organizational inclusion, and microaggressions) and retention-related measures (i.e., job satisfaction, affective commitment, and intentions to leave), the contribution of feelings of inclusion to retention measures, and the effect of numerical representation and number of marginalized identities on these concepts. We analyzed data from the 2022 Your Say Matters survey, which was administered to a representative sample of CAF members, with oversampling of under-represented groups. Respondents included 4,483 Regular Force members (30.9% response rate). The groups under study included Indigenous members, persons with disabilities, racialized members, women not part of another group (non-Indigenous, non-racialized, women without disabilities), and everyone else (non-Indigenous, non-racialized, not women, without disabilities). Our hypotheses were supported overall, such that groups with less representation in the CAF scored lower on inclusion measures than groups with more representation. The number of marginalized identities held by military members predicted the inclusion measures, but did not predict retention-related measures. There were some group differences on retention-related measures, such that women not part of another group scored more favorably than other designated groups, and racialized members scored more favorably than PwD and Indigenous members. Inclusion measures predicted job satisfaction, affective commitment, and intentions to leave equally for all groups studied, suggesting that feeling included is important for all.

## 1 Introduction

The Canadian Armed Forces (CAF) is committed to creating a welcoming, fair and *inclusive* environment for all its members by reducing barriers for designated groups (Canadian Armed Forces Employment Equity Plan 2021-2026, 2022). The CAF is also committed to increasing retention of its members (Department of National Defence, 2022a). Ensuring members are included is one way in which the CAF can increase retention, which would aid in reconstitution efforts (Department of National Defence, 2022b). In this paper, we explore the inclusion experiences of under-represented Regular Force members (i.e., Indigenous members, persons with disabilities, racialized members, women) and describe potential barriers to their inclusion. We suggest that group size will be related to members’ sense of inclusion, such that smaller groups will feel less included than larger groups. We review literature suggesting that when members feel included, they are more likely to remain in their workplace. We then assess whether there are group differences on key retention outcomes (namely, job satisfaction, affective commitment, and intentions to leave). We assess whether inclusion predicts these retention outcomes, and test whether group membership moderates this relationship.

### 1.1 Representation of under-represented groups in the Canadian Armed Forces

The Canadian *Employment Equity Act*^1^ seeks to achieve equality in the workplace, by requiring that federally regulated employers identify and eliminate barriers to the employment of designated groups (women, Indigenous peoples, persons with disabilities, and racialized members), establish short-term, numerical goals to address underrepresentation of these groups based on workforce availability, and institute policies, programs and accommodations in support of these goals. As a federally regulated employer, the Canadian Armed Forces (CAF) is required to identify and eliminate barriers limiting the employment opportunities of racialized members, women, Indigenous peoples, and persons with disabilities. However, due to the CAF’s “principle of Universality of Service (which requires that CAF members must, at all times and under any circumstances, be able to perform any function required of them),” the CAF does not have the same recruitment and retention requirements for persons with a disability if the disability permanently impedes an individual’s ability to meet Universality of Service (Department of National Defence/Canadian Armed Forces Ombudsman, 2022).

As stated in Canada’s Defence Policy, *Strong Secure Engaged* (Department of National Defence, 2017), “the Canadian Armed Forces is committed to demonstrating leadership in reflecting Canadian ideals of diversity, respect and inclusion, including striving for gender equality and building a workforce that leverages the diversity of Canadian society.” The Canadian Armed Forces Employment Equity Plan 2021-2026 (2022)^2^ outlines the CAF’s plan to meet its obligations under the *Employment Equity Act*, as well as its short-and long-term goals for numerical representation. It indicates that both recruitment and retention of under-represented groups are necessary to reach these goals. Furthermore, the CAF Retention Strategy (Department of National Defence, 2022a) recognizes that multiple groups are under-represented in the military and points to the need to dive deeper into the issues affecting under-represented groups, in order to increase their representation and retention in the CAF by addressing barriers to equity that they may experience. This is particularly crucial considering the personnel shortfall that the CAF is currently experiencing and the rebuilding and reconstitution efforts under way to ensure that the CAF has the numbers, structures, and readiness in place to meet its mandate (Department of National Defence, 2022b). Despite the CAF’s commitments to diversity and employment equity, and the significant reconstitution efforts under way, under-representation of designated groups persists.

According to 2021 Canadian census data, the Canadian population consisted of 26.5% racialized groups (Statistics Canada, 2023a), 25.3% when including only Canadians aged 17 and over, whereas only 13.9% of serving CAF members were from racialized groups (Statistics Canada, 2023b). In comparison, only 10.7% of the Regular Force (Reg F) voluntarily self-identified as racialized members in 2023 (Department of National Defence, 2023). Similarly, Canada consisted of 5% Indigenous Peoples in 2021 (Statistics Canada, 2022b), or 4.4% when including only Canadians aged 17 and over. Based on 2021 census data, 5.5% of serving CAF members were Indigenous;^3^ however, internal reports of Indigenous representation in the CAF indicate that only 3.0% of the Reg F voluntarily self-identify as Indigenous (Department of National Defence, 2023).^4^

In addition, 2021 Canadian census data revealed that women represented 50.9% of the Canadian population (Statistics Canada, 2022a), but only 18.7% of active CAF members (Statistics Canada, 2023b). Internal reports indicate that when looking at the Reg F in particular, only 16.1% were women (Department of National Defence, 2023). Finally, in 2017, 22% of Canadians had a disability (Statistics Canada, 2022c), whereas 1.2% of the Reg F and Primary Reserve had a disability in 2023 (Department of National Defence, 2023); this discrepancy is at least partially due to the fact that military universality of service requirements render individuals with certain types of disabilities ineligible to join the CAF [Canadian Human Rights Act (R.S.C., 1985, c. H-6, 2012, c. 16, s. 83), 2012]. Internal reports reveal that progress toward representation goals in the CAF has been slow: the 2021–2022 employment equity report (Department of National Defence, 2022c) shows a slow increase in the representation of Indigenous and racialized military members, and a stagnated rate of increase for women. Despite the CAF’s goal of removing barriers to the recruitment and retention of designated groups, this slow progress suggests that barriers remain.

### 1.2 Barriers to representation and inclusion

Although the CAF collects data from voluntarily releasing members via the CAF Exit Survey (Bremner et al., 2017) to better understand reasons for release, response rates have historically been very low, limiting the reliability and representativeness of results, particularly for smaller sub-groups such as some of the designated groups. Although improvements to survey administration are under way, it is not currently a reliable source of information for under-represented groups.

That being said, results of focus groups and interviews conducted as part of a CAF Employment Systems Review (ESR; Skomorovsky and Lalonde-Gaudreault, 2013) point to several potential barriers to the employment of designated groups. For example, one such barrier is perceived to lie in the career management and training systems; participants perceived that opportunities afforded by these systems were based on popularity contests, putting designated groups at a disadvantage. Participants also reported that the biggest perceived barriers for racialized members were integration-related (adjusting to the CAF culture and language requirements), that family-related concerns were prominent for both women and racialized members, and that harassment and discrimination directed at under-represented groups were also barriers. The 2019 survey research in support of the CAF ESR (Price et al., 2020) also found that racialized members, Indigenous members, persons with disabilities, and women perceived barriers to their career advancement. External reviews have also documented some of the employment barriers that equity-seeking groups face. These include enquiries into sexual assault and harassment in the CAF conducted by Deschamps (2015) and Arbour (2022), and evidence provided by the Heyder and Beattie Class Action lawsuit (Minister of National Defence Before the Standing Committee on National Defence, 2020). Moreover, the Minister of National Defense Advisory Panel on Systemic Racism and Discrimination described examples of systemic racism and discrimination (including anti-Indigenous and anti-Black racism, LGBTQ2+ prejudice, gender bias, right-wing extremism and white supremacy; Minister of National Defence, 2022).

Despite the barriers experienced by these groups, attrition rates for designated groups are similar to those of the overall CAF Reg F population (Straver, 2021, 2022): whereas an attrition rate of 6.4% was observed in 2020/2021 for the Reg F as a whole, attrition rates were higher for Indigenous members (7.1%) and persons with disabilities (6.8%), slightly lower for women (6.3%), and lower for racialized members (4.8%). Nonetheless, reduction of attrition rates, in tandem with higher recruitment rates, is crucial for increasing the representation of designated groups.

### 1.3 The present study

In light of the CAF’s commitment to creating a welcoming, fair and *inclusive* environment for all its members, its commitment to reducing barriers for designated groups (Canadian Armed Forces Employment Equity Plan 2021-2026, 2022), and the urgency of CAF reconstitution efforts, the objective of this study is to examine CAF members’ perceptions of inclusion, the impact of these perceptions on various retention factors, and whether this association varies based on membership in one or more designated groups. Specifically, we examine the perceptions and attitudes of under-represented Regular Force members (i.e., Indigenous members, persons with disabilities, racialized members, women) in terms of their perception of inclusion, and focus on key retention outcomes, as feeling excluded in the workplace can lead to lower retention of members from non-dominant groups. We examined multiple proximal precursors of employee turnover, namely, job satisfaction, affective commitment, and intentions to leave, which have been shown to be some of the best predictors of turnover behavior (Griffeth et al., 2000).

#### 1.3.1 Inclusion

Inclusion in the workplace can be conceptualized at the individual and organizational levels (Shore et al., 2018). According to Jansen et al. (2014), alongside autonomy or authenticity, an individual’s sense of relatedness is an important component of feeling included. Relatedness is defined as an individual’s feeling of connection to a group (Baumeister and Leary, 1995) and as an individual’s experience of communion and close relationships with others (Van den Broeck et al., 2010). Conversely, inclusion can be experienced as the absence of behaviors seeking to exclude; for example, experiences of microaggressions signal to individuals that they are not included in the social environment. Microaggressions are defined as “commonplace verbal or behavioral indignities, whether intentional or unintentional, which communicate hostile, derogatory, or negative racial slights and insults” (Sue et al., 2007, p. 278). Although originally studied in reference to racialized groups, the study of microaggressions has expanded to capture other under-represented groups’ experiences, such as those of women (Basford et al., 2014), persons with disabilities (Olkin et al., 2019), and lesbian, gay, bisexual, transgender, and queer (LGBTQ+) members (Platt and Lenzen, 2013; Resnick and Galupo, 2019). Inclusion can also be experienced at the organizational level, through measuring individuals’ perception that the organization is actively working to include diverse members (Van den Broeck et al., 2010).

Experiences of inclusion are not homogeneous. Tokenism (Kanter, 1977) suggests that the experiences of minority groups in the workplace are a function of their numerical proportion, and that their inability to achieve equality is due to their token status. Critical mass theory (Thomas, 1991) further suggests that when this proportion reaches a certain level, conditions for minority group members will qualitatively shift. Yoder’s (1991) review of research on tokenism suggested that the negative consequences of tokenism only occur for groups that are of a lower status than the majority group, such as gender-based groups, and lower status racial groups; two of these well-documented consequences are social isolation and role encapsulation, both a hinderance to inclusion. For example, Hillard et al. (2014) found a linear relationship between the number of women faculty in STEM departments and women’s experience of discrimination, suggesting that the more members of a disadvantaged group are present in a work environment, the less discrimination women experience. Watkins et al. (2019) conducted a review of both military and non-military research on tokenism and found that both women and racial minorities had more negative work appraisals (including job satisfaction, harassment, and discrimination) when they were tokens, and experienced worse effects when they were the only member of their group in a work setting. Based on Kanter’s theory of tokenism, and on Yoder and Watkins et al.’s reviews, we predict that the more numerically scarce an under-represented group is within the CAF, the less included members of that group will feel.

Research conducted in the CAF further supports the notion that under-represented groups will feel less included than majority-group members. Qualitative research with CAF members suggests that the military culture prioritizes some identities over others. For example, Brown (2020) found that senior officer professional training prioritized masculinity, whiteness, heterosexuality, and combat warrior identities, and George’s (2020) interviews with racialized women in or retired from the CAF revealed that the military warrior is socially constructed as stereotypically male, based on a “white settler” mythology, and excludes those outside of the masculine warrior norm (i.e., women), Indigenous Peoples, and racialized Canadians. When some identities are privileged over others in the workplace, members of non-dominant groups feel less included than majority group members (Cheeks and Yancey, 2022).

Based on the numerical representation of designated groups in the CAF, we would expect Indigenous CAF members and those with disabilities to have the lowest levels of inclusion, followed by racialized CAF members, and women who are not part of another group. CAF members who are not members of a designated group are expected to report the highest levels of inclusion. Although military literature in this area is scarce, these predictions are supported by research with non-military samples. Studies found that women tend to feel less included in the workplace than men (Mor Barak et al., 2001; Mor Barak and Levin, 2002; Findler et al., 2007; Blank et al., 2021), racialized groups are more likely to feel excluded than White individuals (Mor Barak et al., 1998; Mor Barak and Levin, 2002; Blank et al., 2021), and Indigenous individuals are more likely to face individual, organizational, and systemic discrimination than non-Indigenous, non-visible minorities (Cotter, 2022; Durand-Moreau et al., 2022). Finally, persons with a disability are more likely to experience exclusion than those without a disability (Blank et al., 2021; Lindsay et al., 2022, 2023).

*H1.* Designated groups with lower representation in the CAF will feel less included than groups with higher representation.

However, social identities are not unidimensional. Individuals have complex, intersecting identities, and multiple forms of oppression can impact the same individual (Crenshaw, 1989). Research suggests that those who are members of multiple under-represented groups also feel less included. Studies with non-military samples have found that women of color have the least positive inclusion climate perceptions when compared to non-racialized men and women and racialized men (e.g., Mor Barak et al., 1998). African American and Asian American women also experience more institutional and social isolation than White men (Smith and Calasanti, 2005). Although Watkins et al. (2019) indicate that intersectional research on tokenism is scarce, they found, for example, that workers with multiple lower-status identities, such as Black male nurses, experienced more isolation than those with fewer lower-status identities, such as White male nurses. When examining studies in the CAF, qualitative studies found that women with one or more intersecting identities seem to have unique, and often negative, experiences related to inclusion and belonging, such as experiences of discomfort, discrimination, and sexual misconduct (George, 2020; Biskupski-Mujanovic, 2022). Based on these findings, we posit the following:

*H2*. CAF members with multiple marginalized identities will have less positive inclusion scores than those with fewer marginalized identities.

#### 1.3.2 Precursors of retention

Tokenism theory posits that the numerical proportion of groups will not only influence their experiences, but also their behavioral responses to these experiences (Kanter, 1977). Given the CAF’s focus on increasing the representation of designated groups in the CAF, including retaining members of these groups, we focus on proximal precursors of employee turnover behavior. Meta-analyses of employees in both military and civilian contexts (Griffeth et al., 2000; Licklider, 2011; Rubenstein et al., 2017) indicate that job satisfaction, affective commitment (i.e., attachment to the organization; Meyer et al., 1993), and turnover thoughts and intentions, are some of the best predictors of turnover behavior.

CAF studies of designated group differences (other than those focusing on women) are limited (Department of National Defence, 2022a), due in part to small sample sizes and relatively low survey response rates. Research looking at gender differences in the CAF revealed that women had higher job satisfaction than men, although the effect size was small, and had similar levels of affective commitment and turnover intentions as men (Pearce, 2020; Yeung et al., 2020). However, studies in the US military (among dual-service couples, Huffman et al., 2017; in general, Kelty et al., 2010), suggest that women have higher turnover intentions.

Studies of civilian employees with a disability have generated mixed results: studies show that employees with a disability have similar (e.g., Kocman and Weber, 2018) or lower (e.g., Schur et al., 2017) levels of job satisfaction than their colleagues without a disability, similar affective commitment levels (e.g., Schur et al., 2017), and similar (e.g., Schur et al., 2017) or higher (e.g., Chordiya, 2022) turnover intentions than their counterparts. Similarly, studies of racialized employees have generated mixed results: racialized employees are found to have similar (e.g., Fakunmoju, 2020; American Indians and Alaska Natives) or lower levels of job satisfaction (e.g., Koh et al., 2016; meta-analysis, including Black workers), lower (e.g., Ozer, 2020) or higher (e.g., Rupert et al., 2010) levels of affective commitment, and higher turnover intentions (Hofhuis et al., 2014) than their ethnic majority counterparts.

In light of mixed evidence of differences in retention-related factors by gender, disability, and ethnicity, and considering that turnover behavior of designated groups in the Reg F is at similar or lower rates than the Reg F average (Straver, 2021), we assessed whether there were group differences in proximal precursors of retention corresponding to their numerical representation.

*H(3a)*. Designated groups with lower representation in the CAF will have more negative scores on retention-related measures than groups with higher representation.

Furthermore, research examining the combined effects of harassment based on several identities (i.e., gender and ethnicity; Raver and Nishii, 2010) found support for an inurement effect for job satisfaction and organizational commitment, that is, adapting or habituating to the hardships, such that the different harassment types did not have an additive effect on these retention outcomes. As such, we predict that:

*H(3b)*. In support of an inuring effect, the number of marginalized identities will not predict a significant amount of variance in the retention factors.

#### 1.3.3 Association between inclusion and precursors of retention

Increasing the recruitment of traditionally under-represented groups alone will not increase their representation in the workplace if members of these groups do not stay in the organization. For members of under-represented groups to stay in the CAF, they must feel included. Indeed, retention rates of under-represented groups are often used as a measure of how successful an organization has been in supporting diversity (Molefi et al., 2021).

**Figure 1:**
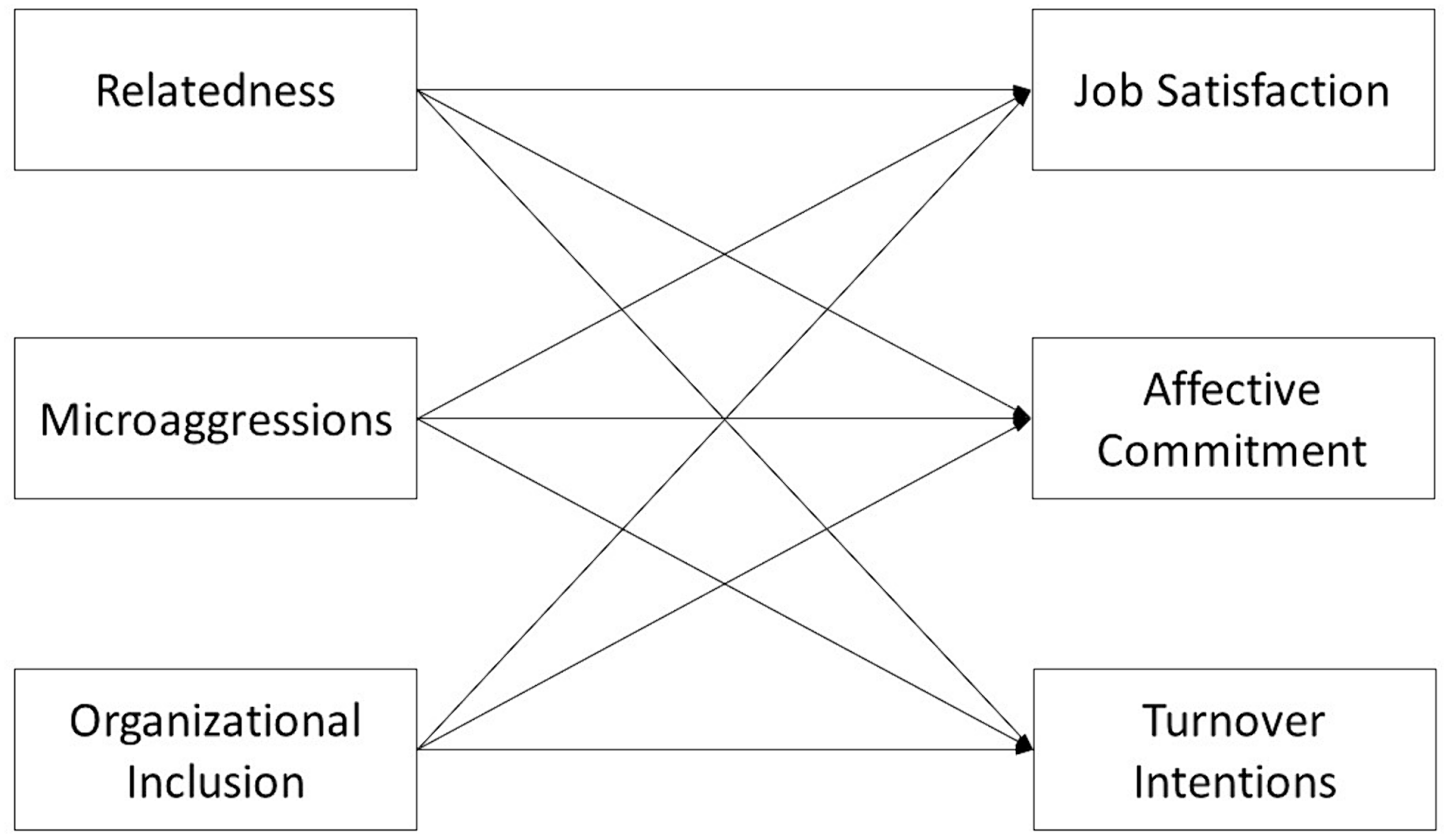
Model depicting Hypothesis 4: Inclusion measures (i.e., relatedness, microaggressions, and organizational inclusion) will predict retention-related measures (i.e., job satisfaction, affective commitment, and turnover intentions).

The more included CAF members feel, the more likely they should be to stay in the organization; however, the contribution of inclusion to retention has been understudied in the military in general, and in the CAF in particular. As such, we turn to civilian studies to inform the expected inclusion-retention associations. Previous research in non-military settings has found that *perceived inclusion* predicts job satisfaction and affective commitment (Mor Barak et al., 2001; Cantarelli et al., 2016; Holmes et al., 2021; Cheeks and Yancey, 2022), including in longitudinal studies (Brimhall et al., 2022), and also predicts turnover intentions (Heera and Maini, 2019). *Relatedness* was found to predict job satisfaction, affective commitment, and intentions to stay in an Italian sample (Colledani et al., 2018). *Microaggressions* were found to predict job outcomes such as job satisfaction and turnover intentions (meta-analysis; Costa et al., 2023), as well as organizational commitment (e.g., Lee, 2009; Jackson and Jackson, 2019). *Organizational inclusion* has been shown to predict lower intentions to leave in a military sample (Merlini et al., 2019) and unit-level turnover in a non-military sample (Nishii, 2013). In light of these findings, we predict that the more included members feel, the more satisfied they will be with their jobs, the more committed they will be to the CAF, and the more likely they will be to intend to stay in the CAF (see Figure 1).

*H4*. Inclusion measures (i.e., relatedness, microaggressions, and organizational inclusion) will predict scores on proximal precursors of retention (i.e., job satisfaction, affective commitment, and turnover intentions).

Finally, although inclusion is beneficial for everyone, it should be especially helpful for those who have been historically excluded (Shore et al., 2018). Inclusion provides individuals with the opportunity to succeed and is present when barriers to performance have been reduced. Therefore, we predict that inclusion constructs will be a stronger predictor of retention-related constructs for under-represented groups.

*H5*. Designated group membership will moderate the inclusion-retention associations, such that these associations will be stronger for under-represented groups.

## 2 Methods

### 2.1 Procedure

In order to test these hypotheses, secondary analyses of select scales from the Your Say Matters (YSM): CAF Well-Being survey were conducted. The YSM was administered electronically to a stratified random sample of the CAF (Reg F and Primary Reserve members), between March and May 2022, in survey respondents’ language of choice (either English or French).^5^ This paper focuses on the Reg F, which had a response rate of 30.9%. Results were weighted by Defence Team organization, occupational authority, rank group, and self-identified designated group member status, to ensure that the estimates were more representative of the population of interest. To increase precision of designated group estimates, designated group members were oversampled.

### 2.2 Participants

In total, 4,483 Reg F members completed the Your Say Matters survey. When examining unweighted responses from those surveyed, most survey respondents were men (70%), followed by women (27.5%), and a small number indicated they were gender diverse (0.7%). With regards to rank, 39.3% were junior non-commissioned members (NCMs), 26.7% were senior NCMs, 18.0% were junior officers, and 15.7% were senior officers. Overall, 6.2% identified as Indigenous, 16.6% identified as a racialized member, 8.9% identified as having a disability, 8.1% identified as a member of a LGBTQ2+ community, and 68.6% of survey respondents were 35 years or older (see Table 1 for more information). Most surveys were completed in English (85.2%). When YSM survey responses were weighted, the percent of Indigenous, racialized, and women in the Reg F were similar to the percent reported by the CAF. This survey estimated that a higher percentage of the Reg F reported a disability (7.5%) than the Reg F reports based on self-identification forms.

**Table 1:** Demographic characteristics.

Demographic characteristic	Reg F population (*N* = 56,928)^a^	Sample unweighted count (unweighted percentage)	Sample weighted percentage
First official language/survey language		4,483	
English	74.4%	3,821 (85.2%)	81.6%
French	25.6%	662 (14.8%)	18.4%
Gender^b^		4,469	
Woman	15.6%	1,228 (27.5%)	16.1%
Man	84.4%	3,128 (70.0%)	81.0%
Gender diverse	–	30 (0.7%)	0.9%.^F,^^d^
Prefer not to disclose	–	83 (1.9%)	2.1%^E,^^e^
Indigenous		4,451	
Yes	3.0%^c^	277 (6.2%)	3.9%
No	–	4,037 (90.7%)	92.4%
Prefer not to disclose	–	137 (3.1%)	3.6%
Member of an ethnicity other than White (racialized member)		4,420	
Yes	10.7%^c^	735 (16.6%)	10.0%
No	–	3,533 (79.9%)	85.6%
Prefer not to disclose	–	152 (3.4%)	4.4%
Person with a disability		4,458	
Yes	1.2%^c^	395 (8.9%)	7.5%
No	–	3,929 (88.1%)	89.1%
Prefer not to disclose	–	134 (3.0%)	3.4%
Member of an LGBTQ2+ community		4,431	
Yes	–	362 (8.2%)	7.1%
No	–	3,890 (87.8%)	87.6%
Prefer not to disclose	–	179 (4.0%)	5.3%
Age		4,474	
24 years and under	9.3%	142 (3.2%)	5.2%
25–34 years	39.2%	1,260 (28.2%)	32.6%
35–44 years	32.1%	1,634 (36.5%)	35.4%
45–54 years	16.0%	1,173 (26.2%)	21.7%
55 years and older	3.5%	265 (5.9%)	5.1%
Rank		4,469	
Junior NCM	53.4%	1,761 (39.4%)	52.5%
Senior NCM	21.9%	1,195 (26.7%)	22.8%
Junior Officer	15.5%	807 (18.1%)	15.3%
Senior Officer	9.2%	706 (15.8%)	9.4%

a This was the sampling frame of the Reg F population used for the YSM study [exclusions = those with less than one year of service, deployed or posted on foreign exchange, on the Basic Training List and the Supplementary Training List, and those on extended leave (e.g., long-term parental leave)].

b Population data represents biological sex (male or female).

c Representation rates as reported in Department of National Defence (2023).

d F refers to estimates with coefficients of variation that were too unreliable to report (i.e., greater than 0.333).

e E refers to estimates with coefficients of variation between 0.166 and 0.333 that should be interpreted with caution.

#### 2.2.1 Prioritized designated group membership

We coded designated group membership such that members who self-identified as belonging to two or more intersecting designated groups were coded into the group with the smallest representation in this sample. The priority order was Indigenous, persons with a disability, racialized members, women not part of another group, and everyone not included elsewhere (i.e., “everyone else”). The everyone else group consisted of those who were not Indigenous, persons with a disability, racialized members, or women.^6^ It included those who had missing data on the demographic questions that assessed gender and designated group membership. It also consisted of the few members who identified as gender diverse, because this group was too small to analyze separately.^7^ We created these groupings to reduce the number of variables needed to capture group differences. Although we were interested in conducting intersectional analyses of gender and the other designated group memberships, we did not have enough survey respondents to examine gender differences among Indigenous, racialized, and disabled CAF members. Thus, this method allowed us to examine women not captured elsewhere (who are mainly White women without disabilities) separately from the everyone else category (who are mainly White men without disabilities).

Although White men are often used as the reference point for comparison, this method of analysis has been criticized by some researchers (e.g., Eichler, 2021) for establishing their experience as the default. Although we do compare members of all designated groups to predominantly White men without disabilities in one of our comparisons, we conducted multiple contrasts to examine group differences between other under-represented groups in the CAF. That is, we compared groups with more representation to groups with less representation in a series of follow-on contrasts, when significant group differences were found.

Among members, 3.9% of the weighted sample were Indigenous, 6.8% were persons with a disability, 9.0% were racialized members, 13.1% were women who did not belong to another group, and 67.1% were not captured elsewhere.^8^

#### 2.2.2 Number of marginalized groups

Although we did not have sufficient sample size to examine the pattern of results for every intersectional identity, we used Lavaysse et al.’s (2018) and Cheeks and Yancey (2022) method to code the number of marginalized identities from each of the under-represented groups into one variable. Members were coded as having a marginalized identity if they indicated they were a person with a disability, Indigenous, a member of a racialized group (i.e., a racialized member), or a woman. Participants received a score of one for every marginalized identity they reported.^9^ Due to a low number of individuals who received the highest possible score of 4 (i.e., less than 10 survey respondents), scores were truncated to 3.

### 2.3 Materials

#### 2.3.1 Relatedness

Relatedness was measured using a 6-item scale adapted from the work-related basic need satisfaction scale (Van den Broeck et al., 2010). Respondents rated their level of agreement with six statements (e.g., “At work, I feel part of a group” and “At work, I can talk with people about things that really matter to me”) on a scale ranging from 1 (*totally disagree*) to 5 (*totally agree*). This scale demonstrated good reliability in this dataset (Cronbach’s α = .88; see Table 2 for correlations between measures).

**Table 2:** Unweighted correlations between inclusion and retention-related measures.

	1	2	3	4	5	6
1. Relatedness	1 (*n* = 4,474)					
2. Microaggressions	−.377** (*n* = 4,466)	1 (*n* = 4,469)				
3. Organizational inclusion	.293** (*n* =4,468)	−.328** (*n* = 4,464)	1 (*n* = 4,476)			
4. Job satisfaction	.460** (*n* = 4,464)	−.258** (*n* = 4,461)	.363** (*n* = 4,466)	1 (*n* = 4,473)		
5. Affective commitment	.371** (*n* = 4,468)	−.175** (*n* = 4,465)	.382** (*n* = 4,469)	.555** (*n* = 4,466)	1 (*n* = 4,475)	
6. Turnover intentions	−.290** (*n* = 3,975)	.196** (*n* = 3,971)	−.305** (*n* = 3,977)	−.583** (*n* = 3,977)	−.570** (*n* = 3,979)	1 (*n* = 3,983)

**p < .001.

#### 2.3.2 Microaggressions

Microaggressions were measured using modified items from Nadal (2011). Specifically, one modified item was from the second-class citizen and assumptions of criminality sub-scale, one was from the microinvalidations sub-scale, and one was from the work or school sub-scale. The other two items were developed in-house. Respondents indicated their degree of agreement with five microaggression experiences (e.g., “Some coworkers make assumptions about me because of my group identity” and “My supervisor treats me differently than other co-workers because of my group identity”). These items were prefaced by the following definition: “Group identity refers to a person’s sense of belonging to a particular group, or to a person’s perception that others believe they belong to a particular group, regardless of whether they actually do or not. Note that a person may have more than one group identity.” Responses were recorded using a scale ranging from 1 (*strongly agree*) to 7 (*strongly disagree*; Cronbach’s α = .85).

#### 2.3.3 Organizational inclusion

Members’ perceptions of organizational inclusion efforts were measured using modified items from Deloitte (2017) and the Government of Australia (2016), and one item was developed in-house. Respondents indicated their level of agreement with three statements (e.g., “The CAF’s leadership has taken meaningful action to help the CAF become a more diverse and inclusive place to work”) on a scale ranging from 1 (*strongly disagree*) to 5 (*strongly agree*; Cronbach’s α = .83).

#### 2.3.4 Job satisfaction

Job satisfaction was measured using three items modified from the Michigan Organizational Assessment Questionnaire (Cammann et al., 1979, 1983), on a scale from 1 (*strongly disagree*) to 5 (*strongly agree*; Cronbach’s *α* = .89). The item “All in all I am satisfied with my job” was replaced by the item “Overall, I am satisfied with my job.”

#### 2.3.5 Affective commitment

Survey respondents rated their affective commitment on a shortened sub-scale from the Organizational Commitment Questionnaire (Meyer et al., 1993). Respondents indicated their degree of agreement with four items (e.g., “I do not feel “emotionally attached” to the CAF”) on a scale ranging from 1 (*strongly disagree*) to 6 (*strongly agree*). The shortened version of the scale demonstrated good internal consistency in the current dataset (Cronbach’s α = .80).

#### 2.3.6 Turnover intentions

A scale by Coralli (1984) was used to measure turnover intentions, which had good internal consistency in this dataset (Cronbach’s α = .80). Respondents rated their agreement with three items (e.g., “I frequently think of quitting my job” and “I am planning to search for a new job during the next 12 months”) on a scale ranging from 1 (*strongly disagree*) to 5 (*strongly agree*).

#### 2.3.7 Data analysis

##### 2.3.7.1 Weighted group comparisons

SPSS Complex Samples was used to analyze weighted responses. We conducted comparisons based on prioritized designated group membership, which was coded so that 1 = everyone not captured in another group, 2 = women not part of another group, 3 = racialized, 4 = persons with disabilities (PwDs), and 5 = Indigenous. The SPSS Complex Samples General Linear Model (CSGLM) was used to conduct ANOVAs to test for group differences. When group differences were found, Helmert contrasts were conducted to compare groups with higher representation to groups with less representation, creating four dummy codes:

W1 = everyone else (i.e., those without a disability, who are not Indigenous or racialized, and are not women) compared to all other groups

W2 = women not part of another group (i.e., women without a disability, who are not Indigenous or racialized) compared to persons with a disability, Indigenous, and racialized members

W3 = racialized compared to persons with disabilities and Indigenous; and

W4 = persons with disabilities compared to Indigenous.

We conducted these comparisons on constructs related to inclusion (relatedness, microaggressions, and organizational inclusion) and constructs related to retention (job satisfaction, affective commitment, and intentions to leave).

We used regression to examine whether survey respondents’ number of marginalized identities was related to inclusion and retention-related measures. We used CSGLM in complex samples,^10^ but used marginalized identities as a continuous rather than a categorical predictor.

**Figure 2:**
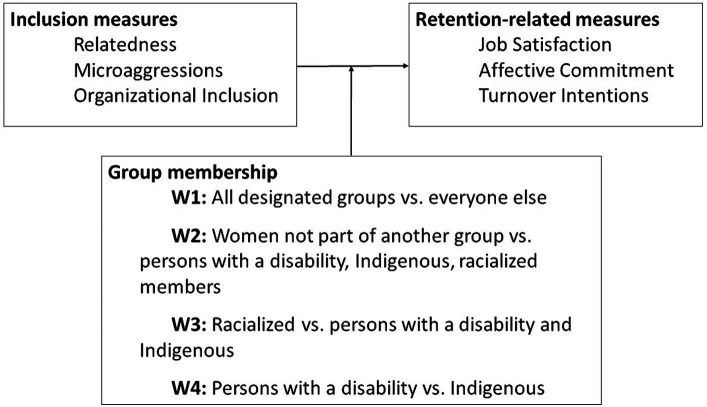
Predicted model: Designated group membership will moderate relationship between inclusion and retention.

We used the PROCESS add-on in SPSS to examine whether designated group membership moderated the relationship between inclusion measures (relatedness, microaggressions, and organizational inclusion) and intention-related measures (job satisfaction, affective commitment, and intentions to leave; see Figure 2). These analyses were not weighted and are available in the Supplementary material.

##### 2.3.7.2 Inclusion measures predicting retention-related measures

We examined the relation between each inclusion-related measure (i.e., relatedness, microaggressions, and organizational inclusion) and each retention-related measure (i.e., job satisfaction, affective commitment, and intentions to leave) using CSGLM in complex samples, again modifying the syntax so that the IV was treated as a continuous variable (using the WITH command).

##### 2.3.7.3 Coefficient of variation

We assessed whether weighted results could be generalized to the population of interest (i.e., the Reg F) using the coefficient of variation (CV). We used Statistics Canada (2009) recommended cutoffs for the interpretation of the CV. Specifically, CVs equal to or less than .166 indicate acceptable sampling variance and thus, estimates were deemed reliable; estimates with CVs between .166 and .333 were interpreted with caution; and estimates with CVs greater than .333 were not reported because they had unacceptably high sampling variance and were deemed unreliable.

##### 2.3.7.4 Effect size

In this paper, we use *R^2^* to report the percent of variance explained in the dependent variable by the independent variable. Using Gignac and Szodorai’s (2016) recommendations, we interpret small, medium, and large effect sizes using percentiles from social and personality psychology, based on meta-analyses of effect sizes. Specifically, we interpret correlations of .11 (*R*^2^ of .0121), .19 (*R*^2^ of .0361), and .29 (*R*^2^ of .0841) as small, medium, and large effect sizes, respectively.

## 3 Results

### 3.1 Group differences in inclusion measures

**Figure 3:**
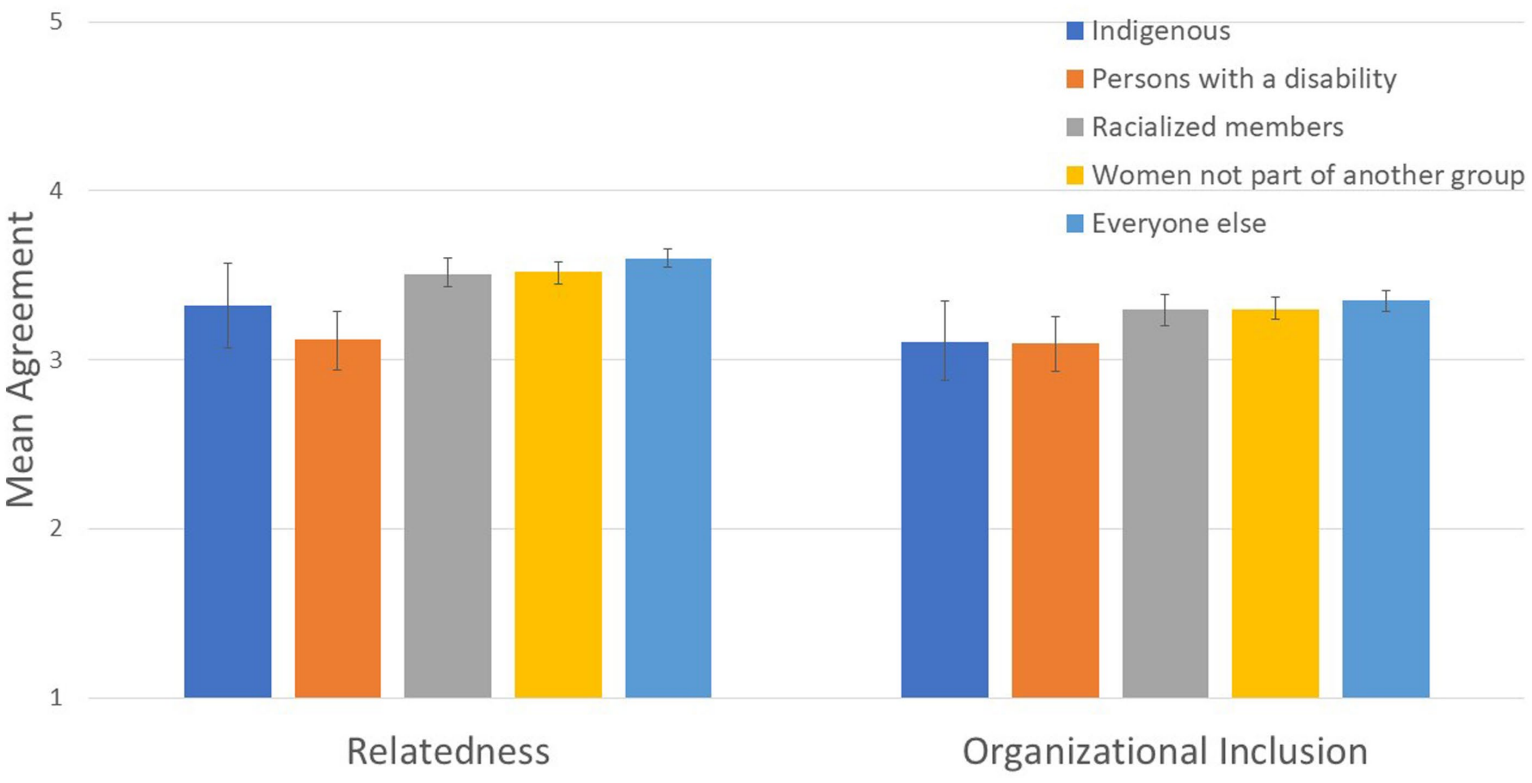
Designated group differences in relatedness and organizational inclusion.

**Figure 4:**
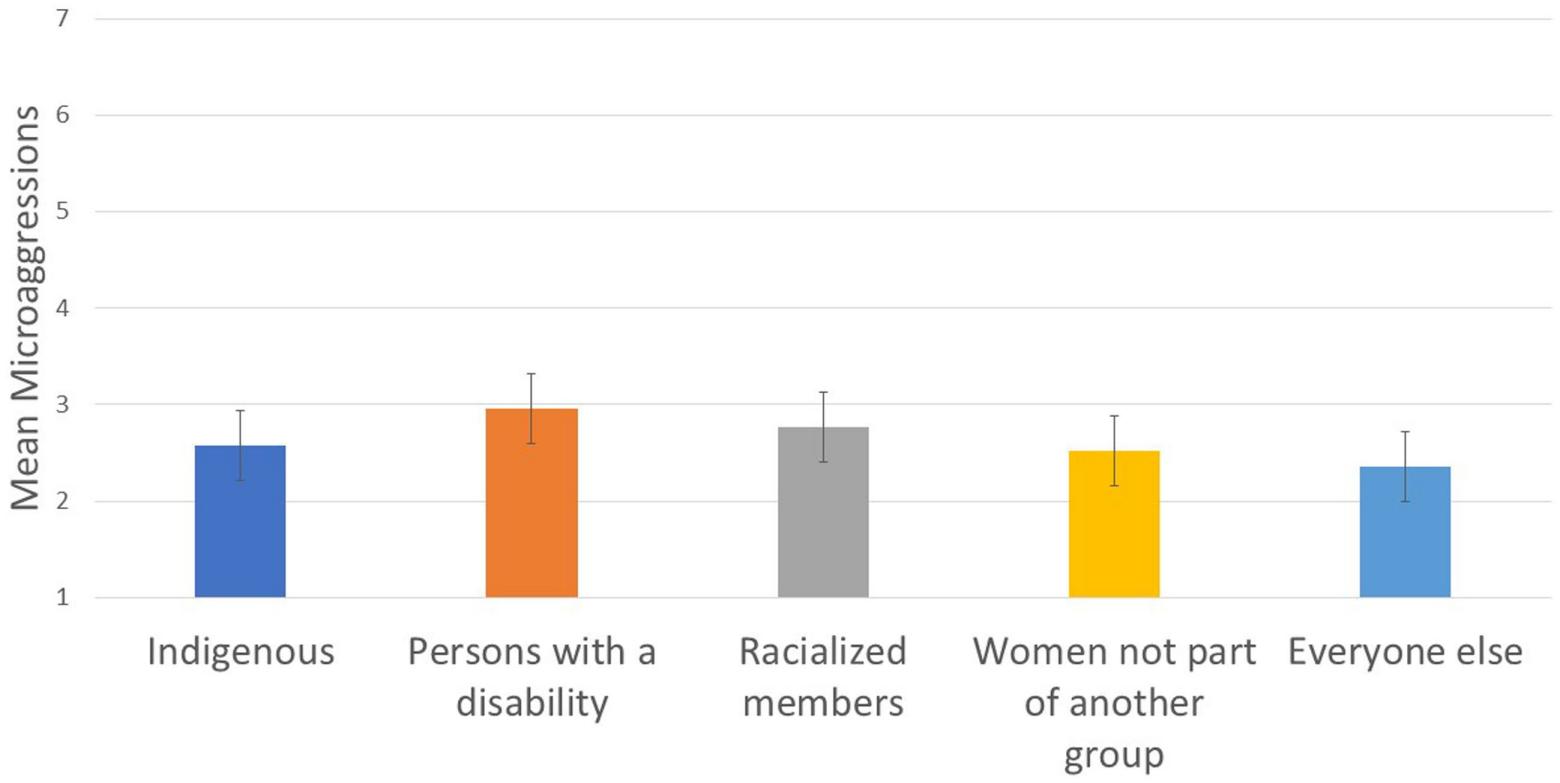
Designated group differences in microaggressions.

To assess Hypothesis 1, we tested whether groups with higher representation reported more positive inclusion (assessed using relatedness, microaggressions, and organizational inclusion) than groups with lower representation. We found group differences on relatedness (*R*^2^ = .024; see Figure 3), organizational inclusion (although it was a small effect size, *R*^2^ = .007; see Figure 3), and microaggressions (*R*^2^ = .019; see Figure 4 and Table 3).

**Table 3:** Estimated marginal means, confidence intervals, and ANOVA results.

Variable	Indigenous	Persons with a disability	Racialized members	Women not part of another group	Everyone else			
	Mean [95% CI]	Mean [95% CI]	Mean [95% CI]	Mean [95% CI]	Mean [95% CI]	*Wald F*	*p*	*R*^2^
Relatedness	3.32 [3.07, 3.57]	3.12 [2.94, 3.29]	3.51 [3.43, 3.60]	3.52 [3.45, 3.58]	3.60 [3.55, 3.66]	7.67	< .001	.023
Microaggressions	2.58 [2.22, 2.94]	2.96 [2.70, 3.21]	2.77 [2.60, 2.94]	2.52 [2.42, 2.62]	2.36 [2.28, 2.45]	8.03	< .001	.019
Organizational inclusion	3.11 [2.88, 3.35]	3.10 [2.93, 3.26]	3.30 [3.20, 3.39]	3.30 [3.24, 3.37]	3.35 [3.29, 3.41]	2.64	.032	.007
Job satisfaction	3.35 [3.09, 3.60]	3.10 [2.91, 3.29]	3.59 [3.44, 3.74]	3.65 [3.57, 3.73]	3.47 [3.39, 3.54]	8.86	< .001	.013
Affective commitment	3.19 [2.88, 3.50]	3.26 [3.06, 3.47]	3.76 [3.65, 3.88]	3.75 [3.67, 3.83]	3.55 [3.47, 3.63]	9.04	< .001	.013
Turnover intentions	2.68 [2.40, 2.95]	2.96 [2.73, 3.18]	2.58 [2.44, 2.71]	2.37 [2.28, 2.47]	2.59 [2.51, 2.68]	6.95	< .001	.013

Relatedness: Women, racialized members, Indigenous members, and persons with disabilities reported lower relatedness than the everyone else group, Wald *F* (1, 4,167) = 22.433, *p* < .001. Women not part of another group reported higher relatedness than other designated groups (i.e., racialized members, Indigenous members, and persons with disabilities), Wald *F* (1, 4,167) = 10.09, *p* = .002. Persons with disabilities and Indigenous members reported lower relatedness than racialized members, Wald *F* (1, 4,167) = 11.15, *p* = .001.

Microaggressions: Women and other designated groups reported more microaggressions than the everyone else group, Wald *F* (1, 4,162) = 20.737, *p* < .001. Women not part of another group reported less microaggressions than other designated groups, Wald *F* (1, 4,162) = 6.656, *p* = .01.

Organizational inclusion: Women and other designated groups reported lower organizational inclusion than the everyone else group, Wald *F* (1, 4,169) = 7.962, *p* = .0005. Women not part of another group reported higher organizational inclusion than the other designated groups, Wald *F* (1, 4,169) = 4.709, *p* = .03. Racialized members reported higher organizational inclusion than persons with disabilities and Indigenous members, Wald *F* (1, 4,169) = 4.616, *p* = .032.

### 3.2 Number of marginalized identities predicting inclusion and retention measures

In line with Hypothesis 2, the number of marginalized identities predicted relatedness (*R*^2^ = .012), microaggressions (*R*^2^ = .014), and organizational inclusion (*R*^2^ =.005), such that members with more marginalized identities experienced less inclusion (see Table 4). It is important to note, however, that marginalized identities explained only a small amount of variance in relatedness and organizational inclusion.

**Table 4:** Regression results: number of marginalized identities predicting inclusion measures.

Variable	Estimate	95% CI	*t*	*p*	*R*^2^
Relatedness	−0.161	−0.223, −0.098	−5.01	< .001	.012
Microaggressions	0.273	0.175, 0.370	5.47	< .001	.014
Organizational inclusion	−0.111	−0.179, −0.043	−3.19	.001	.005

### 3.3 Group differences in retention measures

**Figure 5:**
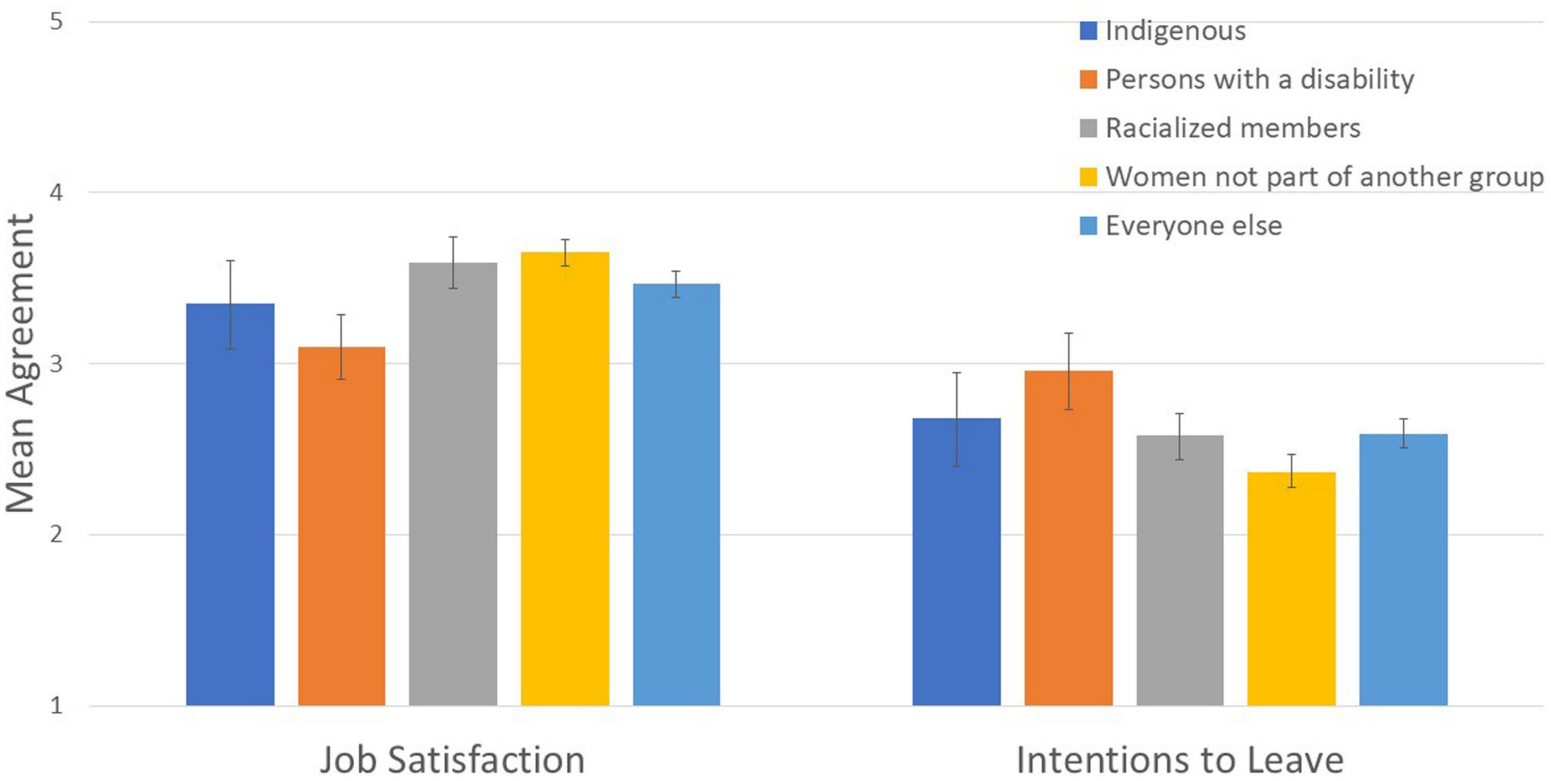
Designated group differences in job satisfaction and intentions to leave.

**Figure 6:**
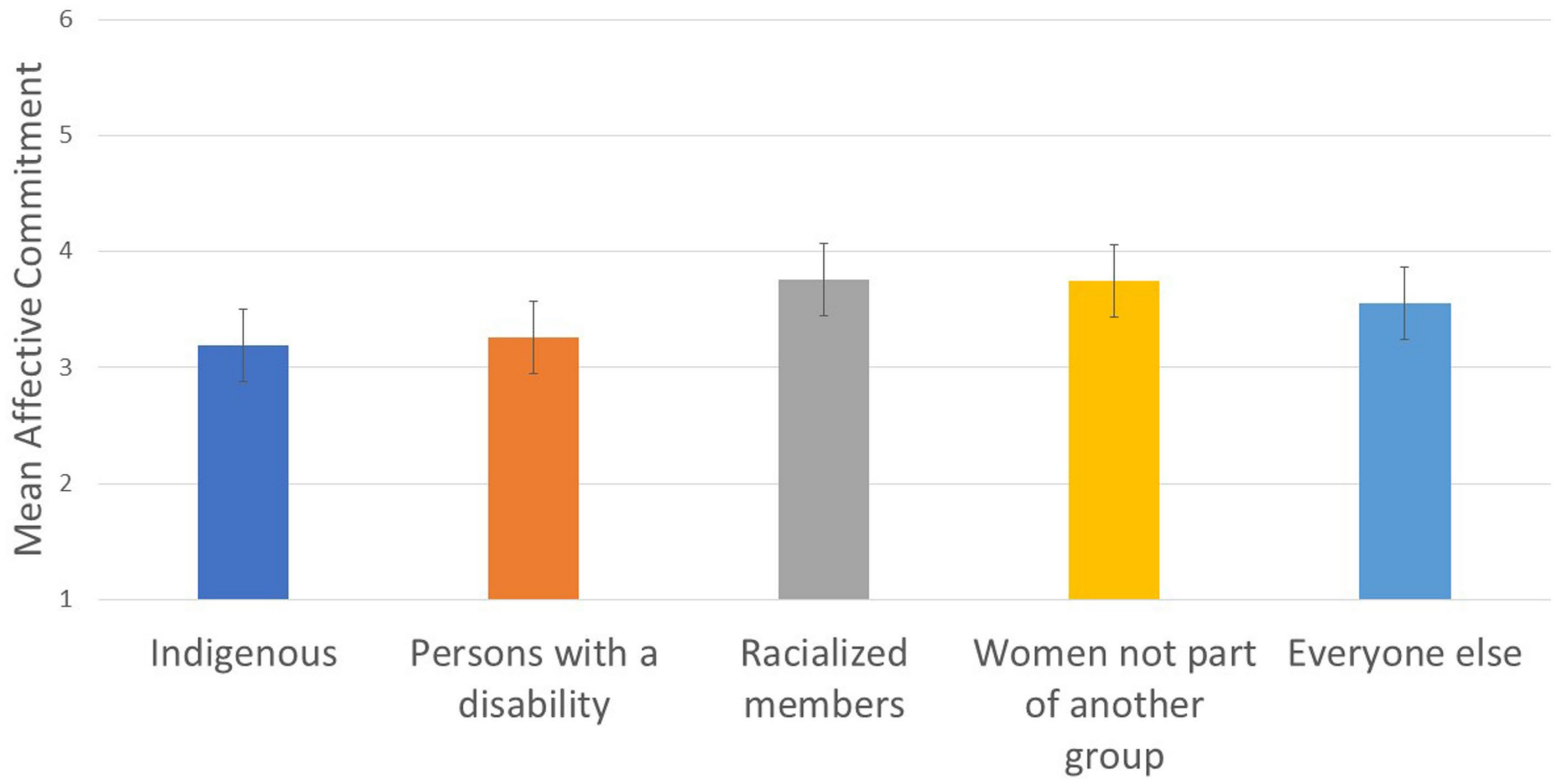
Designated group differences in affective commitment.

To test Hypothesis 3a, we next explored whether groups with higher representation reported more positive scores on proximal precursors of retention (job satisfaction, affective commitment, and intentions to leave) than groups with lower representation. We found small group differences on job satisfaction (*R*^2^ *=*.013; see Figure 5), intentions to leave (*R*^2^ = .013; see Figure 5), and affective commitment (*R*^2^ = .013; see Figure 6 and Table 3).

Job satisfaction: Women not part of another group reported higher job satisfaction than other designated groups, Wald *F* (1, 4,166) = 18.783, *p* = .0001, and racialized members reported higher job satisfaction than PwD and Indigenous members, Wald *F* (1, 4,166) = 11.225, *p* = .001.

Affective commitment: Women not part of another group reported higher affective commitment than other designated groups, Wald *F* (1, 4,168) = 19.082, *p* = .0001, and racialized members reported higher affective commitment than PwD and Indigenous members, Wald *F* (1, 4,168) = 22.610, *p* = .0001.

Intentions to leave: Women not part of another group had lower intentions to leave than other designated groups, Wald *F* (1, 3,682) = 20.525, *p* = .0001, and racialized members had lower intentions to leave than PwD and Indigenous members, Wald *F* (1, 3,682) = 4.415, *p* = .036.

### 3.4 Number of marginalized identities predicting retention measures

To test Hypothesis 3b, we assessed whether the number of marginalized identities predicted job satisfaction, affective commitment, and intentions to leave, and found that none of these predictions were significant.

### 3.5 Relation between inclusion measures and retention-related measures

Consistent with Hypothesis 4, all of the inclusion measures (relatedness, microaggressions, organizational inclusion) predicted all of the retention-related measures (job satisfaction, Table 5; affective commitment, Table 6; intentions to leave, Table 7) with microaggressions explaining the least amount of variance in each measure. Specifically, relatedness (*R*^2^ = .184), microaggressions (*R*^2^ = .062), and organizational inclusion (*R*^2^ = .125) predicted job satisfaction. Relatedness (*R*^2^ = .122), microaggressions (*R*^2^ = .035), and organizational inclusion (*R*^2^ = .169) also predicted affective commitment. Finally, relatedness (*R*^2^ = .08), microaggressions (*R*^2^ = .035) and organizational inclusion (*R*^2^ = .092) predicted intentions to leave.

**Table 5:** Regression results: inclusion measures predicting job satisfaction.

Variable	Estimate	95% CI	*t*	*df*	*p*	*R*^2^
Relatedness	.546	.488, .604	18.313	4,159	< .001	.184
Microaggressions	−.203	−.243, −.163	−10.014	4,156	< .001	.062
Organizational inclusion	.430	.366, .493	13.348	4,161	< .001	.125

**Table 6:** Regression results: inclusion measures predicting affective commitment.

Variable	Estimate	95% CI	*t*	*df*	*p*	*R*^2^
Relatedness	.510	.439, .581	14.019	4,163	< .001	.122
Microaggressions	−.176	−.220, −.132	−7.87	4,160	< .001	.035
Organizational inclusion	.573	.507, .639	16.934	4,161	< .001	.169

**Table 7:** Regression results: inclusion measures predicting turnover intentions.

Variable	Estimate	95% CI	*t*	*df*	*p*	*R*^2^
Relatedness	−.380	−.447, −.312	−11.027	3,675	< .001	.08
Microaggressions	.162	.114, .210	6.647	3,671	< .001	.035
Organizational inclusion	−.389	−.457, −.321	−11.269	3,677	< .001	.092

### 3.6 Group moderation between inclusion and retention measures

The results of Hypothesis 5 can be found in the Supplementary material. Although there were some significant interactions between inclusion measures and designated group membership, these interactions always explained less than 1.2% of the variance in the intention-related measure or they were non-significant.

## 4 Discussion

### 4.1 Inclusion

We hypothesized that groups with higher representation in the CAF would feel more included than groups with lower representation. In this study, we used more nuanced comparisons than typical group comparison research. Specifically, we conducted multiple comparisons between more represented and less represented groups to avoid using White men as the reference point for all comparisons. This hypothesis was mainly confirmed. The group composed predominantly of White men reported higher relatedness scores and sense of organizational inclusion than all other groups, and reported lower microaggressions scores. Compared to all other designated groups, women not part of another group reported higher relatedness and organizational inclusion and lower microaggressions scores. These results are consistent with critical mass theory, and suggest that (mainly White) women may have reached a certain threshold in representation in the CAF whereby they experience more favorable outcomes than groups of lesser representation. Women not part of another group, however, had less favorable outcomes than men who were not a member of a designated group. These results are consistent with previous research with non-military employees, which found that women tend to feel less included in the workplace than men (Mor Barak et al., 2001; Mor Barak and Levin, 2002; Findler et al., 2007; Blank et al., 2021), particularly in stereotypically male domains (e.g., Hillard et al., 2014).

Compared to PwD and Indigenous members, racialized members had higher relatedness and organizational inclusion scores; however, inconsistent with our hypothesis, racialized members did not differ from PwD and Indigenous members on microaggressions. Although the trend observed for mean scores (see Table 3) suggest that PwD may experience slightly more microaggressions than Indigenous and racialized members (which may be explained by a focus on Universality of Service in the CAF), the differences did not reach significance, due to lower power for smaller groups. Overall, these group differences are consistent with tokenism (Kanter, 1977; Yoder, 1991; Watkins et al., 2019) and critical mass theory (Thomas, 1991), in that less represented groups had a lower sense of relatedness and organizational inclusion, and experienced more microaggressions, than groups with higher representation.

Inconsistent with our hypothesis, there were no significant differences between Indigenous members and persons with disabilities in relatedness, experiences of microaggressions, and organizational inclusion. Indigenous members and persons with disabilities are both similarly under-represented in the CAF and thus both groups may experience tokenism, which may explain the lack of significant differences between these groups. Unlike previous research on exclusion experienced by Indigenous members (Cotter, 2022; Durand-Moreau et al., 2022) and persons with a disability (Blank et al., 2021; Lindsay et al., 2022, 2023), this research compared both groups to each other rather than to the majority group.

By measuring and comparing designated groups’ sense of inclusion in the CAF, this study adds to our understanding of barriers experienced by designated group members, including barriers in the career management and training systems (Skomorovsky and Lalonde-Gaudreault, 2013; Price et al., 2020), difficulties integrating, family-related concerns, harassment and discrimination (Skomorovsky and Lalonde-Gaudreault, 2013), sexual assault and harassment (Deschamps, 2015; Arbour, 2022), and systemic racism and discrimination (Minister of National Defence, 2022). Although estimated mean scores of inclusion (Table 3) suggest that there is room for improvement for all groups in this regard, results reveal that some designated groups experience inclusion and exclusion differently and at different levels, and indicate that overall, representation is a contributing factor in fostering an inclusive and welcoming workplace and culture for designated groups in the CAF.

Hypothesis 2 was supported. The number of marginalized identities predicted all of the inclusion measures (relatedness, microaggressions, and organizational inclusion). Traditionally, qualitative research (e.g., Brown, 2020; George, 2020; Biskupski-Mujanovic, 2022) has more fully documented intersectional experiences than quantitative research (Watkins et al., 2019), although some quantitative intersectional research has documented the experiences of racialized women (Mor Barak et al., 1998; Smith & Calasanti, 2005). It has traditionally been more difficult to reflect intersectional experiences using quantitative research, as members of non-dominant groups are less represented in the workforce, and therefore, fewer members of these groups complete surveys. This means that statistical analyses can be under-powered to report intersectional experiences. This paper follows the work of Cheeks and Yancey (2022) and Lavaysse et al. (2018) to measure the number of minority identities held by survey respondents and finds that the more under-represented groups a member belongs to, the less included they feel. Although this method does not reflect the lived experience of specific intersectional identities, as advocated for by Crenshaw (1989), it does allow for some intersectional analyses of members’ experiences. These results suggest that some members may feel less included than others, and may be particularly vulnerable to systemic barriers associated with exclusion, especially as they relate to career management and training systems and opportunities, that are seen by members as being influenced by “popularity contests” (Skomorovsky and Lalonde-Gaudreault, 2013).

### 4.2 Precursors of retention

Results revealed more group differences on measures of inclusion than on measures of proximal precursors of retention, which is consistent with previous literature. Our analyses found that there were no differences between designated groups and the everyone else group (predominantly White men) on job satisfaction, affective commitment, and intentions to leave. In support of Hypothesis 3, we found that women not part of another group had higher job satisfaction, affective commitment, and lower intentions to leave than other designated groups. This analysis allowed for a unique contrast that has not been conducted previously, in which we compared White women to other designated groups. Previous research in the CAF (Pearce, 2020; Yeung et al., 2020) and the US military (Kelty et al., 2010; Huffman et al., 2017) have tended to compare women to men.

We also found that racialized members had higher job satisfaction, affective commitment, and lower intentions to leave than PwD and Indigenous members. This is the first comparison of its kind for CAF members. Consistent with our inclusion findings, there were no significant differences between Indigenous members and persons with disabilities in job satisfaction, affective commitment, and intentions to leave. By demonstrating that groups with lower representation in the CAF have lower intentions to stay in the CAF, this study helps to add to our understanding of factors that are related to retention in a military context (Licklider, 2011; Pearce, 2020; Yeung et al., 2020). We recommend that future research examine the unique barriers to retention experienced by each equity-seeking group. For example, women and Indigenous persons may be more adversely impacted by relocation requirements, in light of the prominence of family concerns among these groups (Skomorovsky and Lalonde-Gaudreault, 2013). Although the CAF reports attrition rates for each designated group, we recommend that future research examine whether equity-seeking groups have differential rates of voluntarily releasing from the CAF.

Our analyses found that the number of marginalized identities did not predict retention-related measures (job satisfaction, affective commitment, and intentions to leave). This null effect is consistent with previous findings, in which there was no additive effect of multiple forms of harassment on job satisfaction and organizational commitment (Raver and Nishii, 2010), and is consistent with the similar attrition rates for designated and non-designated group members (Straver, 2021). Although we did not find significant differences in this study, examining whether the number of marginalized identities predicts retention factors is an important contribution to an under-studied domain. Recruitment and retention statistics in the CAF have not consistently measured intersectional identities. We recommend that CAF recruitment and retention rates be examined by intersectional identities when possible, recognizing that not all members complete self-identification forms and some groups may be too small to produce reliable estimates. Conducting intersectional analyses when feasible would add to our understanding of how identifying with more than one under-represented group impacts perceptions of inclusion and retention-related outcomes.

### 4.3 Association between inclusion and precursors of retention

Hypothesis 4 (as depicted in Figure 1) was supported. All of the inclusion measures predicted all of the retention-related measures. These predictions were conducted without controlling for multiple inclusion measures. These results are consistent with previous findings from non-military samples, which found that organizational inclusion predicted job satisfaction and affective commitment (Brimhall et al., 2022 [longitudinal study]; Mor Barak et al., 2001; Cantarelli et al., 2016; Holmes et al., 2021; Cheeks and Yancey, 2022) and turnover intentions (Heera and Maini, 2019), that relatedness predicts job satisfaction, affective commitment, and intentions to stay (Colledani et al., 2018), that microaggressions predict job outcomes (Costa et al., 2023) and organizational commitment (e.g., Lee, 2009; Jackson and Jackson, 2019), and that organizational inclusion predicts unit-level turnover (Nishii, 2013). Moreover, these results add to Merlini and colleague’s et al. (2019) efforts to document the link between organizational inclusion and intentions to leave in a military sample. It is interesting to note that the effect size for inclusion measures predicting retention-related measures was larger than many of the effect sizes for group differences in inclusion and retention measures, suggesting that inclusion may matter more for retention than group membership itself.

We tested whether group membership moderated the relation between inclusion and retention measures in hypothesis 5, such that the association between inclusion experiences and retention-related outcomes was strongest among groups with less representation. Inconsistent with Shore et al. (2018) predictions, results (see Supplementary material) revealed some significant, albeit small, effects. This suggests that inclusion is related to retention for both majority and minority group members, indicating that the moderation model depicted in Figure 2 does not best reflect the data. Although the group membership moderation had a small effect size, we recommend that future research continue to examine the unique characteristics and experiences of equity-seeking groups as they relate to retention, in support of CAF Reconstitution. For example, future research could examine the intersection between group membership and other demographic characteristics, such as age, tenure, rank, gender identity, and sexual orientation.

### 4.4 Limitations and future directions

This study has some limitations. First, inclusion and retention measures were collected at the same time, preventing our ability to determine whether inclusion impacts intentions to leave over time. Although there has been a longitudinal study on the impact of inclusion on job satisfaction and affective commitment (Brimhall et al., 2022), we recommend that this be replicated in a military context. Second, the response rates for this survey were low. Although designated groups were oversampled to increase representation in the sample and results were weighted so that they were representative of the Reg F, non-response remains an important source of bias. Third, members who identified as multiple designated groups were coded into the smallest designated group in this sample. Although we also examined the number of marginalized identities held by each survey respondent, this coding method artificially categorizes a respondent’s identity in a way that oversimplifies the complexity of their identity. Our first category included everyone not captured elsewhere, including those who chose not to self-identify, which also adds some bias to our comparisons. Fourth, it should be noted that individuals were placed into broad, homogenous categories (i.e., Indigenous individuals, persons with a disability, racialized groups). However, in reality, each group is heterogeneous with different histories, cultures, and experiences (e.g., the Indigenous group consists of those who identify as First Nations, Métis, or Inuit). Finally, although we examined results by employment equity groups, there are many other ways in which someone could be under-represented in a military context, such as sexual orientation. Given the CAF’s history of excluding LGBTQ+ individuals [i.e., the purge, Deloitte (2018)] and current efforts to include these individuals, studying inclusion among these respondents would be a timely, important future direction for this field of research. We believe that, given the unique experience of discrimination based on sexual orientation and gender identity in the military, these topics warrant their own examination. We encourage future research to continue to examine under-represented groups’ sense of inclusion and its relation to retention in a military context.

## 5 Conclusion

The CAF is committed to diversity, respect, and inclusion (Department of National Defence, 2017), and is striving to meet its obligations under the *Employment Equity Act* (Department of National Defence Canadian Armed Forces Employment Equity Plan 2021-2026, 2022). Understanding the experiences of under-represented groups plays a small part in this larger objective. This study found that under-represented groups often feel less included in the CAF: they feel less connected to their peers, they experience more microaggressions, and they believe the CAF is doing less to promote inclusion. Among all groups, military members’ sense of inclusion is related to their intention to leave and to other proximal precursors of turnover, namely job satisfaction, and affective commitment. Creating an inclusive environment for all members (Canadian Armed Forces Employment Equity Plan 2021-2026, 2022) will help the CAF move closer to its goal of retaining members of under-represented groups (Department of National Defence, 2022a). To foster greater feelings of inclusion, Shore et al. (2018) suggest that psychological safety, feeling involved in the workplace, feeling respected and valued, having influence over decision-making, feeling like authenticity is supported in the workplace, and feeling that diversity is recognized, honored, and advanced will all increase employees’ perceived inclusion. Ensuring that all members of the CAF feel included will facilitate the retention of more personnel from all groups, which is particularly critical for the CAF in a period of reconstitution (Department of National Defence, 2022b).

## Data availability statement

The data analyzed in this study is subject to the following licenses/restrictions: dataset may contain identifiable information, and thus is not provided. Requests to access these datasets should be directed to Jennifer.Peach@forces.gc.ca.

## Ethics statement

The studies involving humans were approved by the Department of National Defence/Canadian Armed Forces Social Science Research Review Board (SSRRB). The studies were conducted in accordance with the local legislation and institutional requirements. The participants provided their written informed consent to participate in this study.

## Author contributions

JP: Conceptualization, Formal analysis, Methodology, Writing – original draft, Writing – review & editing. JL: Data curation, Project administration, Writing – original draft, Writing – review & editing. KB: Formal analysis, Methodology, Writing – original draft, Writing – review & editing.

## Footnotes

^1^Although the Employment Equity Act refers to Aboriginal peoples and visible minorities, we refer to these groups as Indigenous and racialized based on updated naming conventions for the Government of Canada.

^2^The current Employment Equity Act requires internal representation of employment equity groups to be commensurate with labor participation rather than the overall population. The CAF also has unique eligibility criteria (e.g., citizenship, language proficiency), and includes occupational categories not present in the overall labor market. The Canadian Armed Forces Employment Equity Plan 2021-2026 (2022) includes more information about the calculation of labor market availability and goals for numerical representation.

^3^Statistics Canada’s estimates are based on a census of all Canadians (including military and non-military Canadians). Statistics Canada indicated that the census counted more currently serving members (101,050) than the CAF counted (97,625) in 2020 when the census was conducted. The different methods of counting individuals as currently serving in the Reg F or P Res may explain the differing numbers between the CAF’s calculation and the census calculation.

^4^The CAF estimates the prevalence of EE groups based on members’ self-identification in forms submitted to the CAF. Previous audits indicated some members do not self-identify to the CAF because they fear potential discrimination, want to be seen as part of the group, or are unsure about how their self-identification will be used (Department of National Defence/Canadian Armed Forces Ombudsman, 2022).

^5^Previous research has demonstrated the invariance of the English and French measures (Blais et al., 2022).

^6^Among those not captured elsewhere, 88.3% were men who were not racialized or Indigenous and did not have a disability, 0.6% were not racialized or Indigenous gender diverse individuals with no disability, and 11.2% preferred not to disclose any designated group identity, and/or had missing data on all of the designated group identities.

^7^In total, 30 survey respondents indicated they were gender diverse. Of these 30 survey respondents, 17 were captured in another category (Indigenous, PwD, or racialized), and 13 were captured in the “everyone else” category. Results remained the same when these 13 survey respondents were excluded from analyses.

^8^We examined the weighted ages of each designated group, and found that equity-seeking groups were similar in age to the everyone else category, except that persons with disability are more likely to be older. This may be because members have developed physical disabilities while serving, and they may be receiving medical treatment or being assessed to see if they still meet Universality of Service requirements or will be medically released. Although there is a significant relation between age and group membership, *χ*^2^(11.008, 45993.429) = 65.559, Adjusted *F* = 2.074, *p* = 0.019, most of the cells had overlapping confidence intervals. When confidence intervals did not overlap, everyone else (33.8%) were more likely to be 25–34  years old than persons with disabilities (21.3%).

^9^Results remained the same when gender diversity was also counted as a marginalized identity.

^10^We modified the syntax so that the independent variable was used as a continuous predictor rather than a categorical predictor (e.g., modifying the BY command to WITH, such as CSGLM jobsat WITH relatedness; Zou et al., 2019).
