# Supplementary material for: Employment equity groups’ experience of inclusion and commitment to the CAF
Source: Front Psychol. 2024 May 15;15:1323474. doi: 10.3389/fpsyg.2024.1323474 (PMC11135602; doi:10.3389/fpsyg.2024.1323474)
Supplement: Supplementary file 1 [file Data_Sheet_1.docx]

Supplementary Material

# Supplementary Data

## Moderations

We examined whether designated group membership moderated the relationship between each inclusion measure (relatedness, microaggressions, and organizational inclusion) and each retention-related measure (job satisfaction, affective commitment, and intentions to leave) using PROCESS (see Figure 2). Helmert contrasts were conducted to compare groups with higher representation to groups with less representation, creating four dummy codes:

W1 = everyone else (i.e., those without a disability, who are not Indigenous or racialized, and are not women)^[[1]](#footnote-1)^ compared to all other groups;

W2 = women not part of another group (i.e., women without a disability, who are not Indigenous or racialized) compared to persons with a disability, Indigenous, and racialized members;

W3 = racialized compared to persons with disabilities and Indigenous; and

W4 = persons with disabilities compared to Indigenous.

**Figure 2.** Moderation of inclusion-retention associations by designated group.

### Relatedness and job satisfaction

The overall interaction between relatedness and designated group predicting job satisfaction was not significant, *F* < 1. The specific interactions were also not significant.

### **Microaggressions and job satisfaction**

The overall interaction between microaggressions and designated group predicting job satisfaction was non-significant, Wald *F*(4, 4451) = 1.2699, *p* = .2794, *R^2^_change_* = .0010. The interaction between microaggressions and W1 (i.e., persons with a disability/Indigenous/racialized members/women not part of another group vs. everyone else) was marginally significant, *t* = -1.7173, *p* = .0860, such that the relationship between microaggressions and job satisfaction was stronger for designated groups than everyone else.

### Organizational inclusion and job satisfaction

The overall interaction between organizational inclusion and designated group predicting job satisfaction was marginally significant, Wald *F*(4, 4456) = 2.0575, *p* = .0837, although the interaction explained less than 1% of the variance in job satisfaction, *R^2^_change_* =.0016. The interaction between organizational inclusion and W2 (i.e., persons with a disability/Indigenous/racialized members vs. women not part of another group) was significant, *t* = 2.8460, *p* = .0044, such that there was a weaker relationship between organizational inclusion and job satisfaction for women not part of another group compared to all other groups.

### Relatedness and affective commitment

The overall interaction between relatedness and designated group was not significant, Wald *F*(4, 4458) = 1.4611, *p* = .2113, *R^2^_change_* = .0011; however, the interaction between relatedness and W4 (i.e., persons with a disability vs. Indigenous members) was significant, *t* = 2.1505, *p* = .0316, such that relatedness was a stronger predictor of affective commitment for Indigenous members than for PwD.

### Microaggressions and affective commitment

The interaction between microaggressions and designated group was not significant, (Wald *F* < 1 for the overall interaction and *t* < 1 for the specific interactions).

### Organizational inclusion and affective commitment

The overall interaction term between organizational inclusion and designated group was marginally significant, Wald *F*(4, 4459) = 1.9723, *p* = .0959, *R^2^_change_* = .0015, but none of the interaction terms were significant.

### Relatedness and intentions to leave

There was a significant interaction between relatedness and designated group membership, Wald *F*(4, 3965) = 3.1426, *p* = .0137, although it explained less than 1% of the variance in intentions to leave, *R^2^_change_* = .0029. The interaction between relatedness and W4 (i.e., persons with a disability vs. Indigenous members) was significant, *t* = -2.5890, *p* = .0097, such that relatedness was a stronger predictor of intentions to leave for Indigenous members than for PwD.

### Microaggressions and intentions to leave

The interaction between microaggressions and designated group was not significant, Wald *F*(4, 3961) = 1.9822, *p* = .10*, R^2^_change_* = .0019. The interaction between microaggressions and W1 (i.e., persons with a disability/Indigenous/racialized members/women not part of another group vs. everyone else) was marginally significant, *t* = 1.8250, *p* = .0681, such that microaggressions were a stronger predictor of intentions to leave for designated groups than for everyone else. The interaction between microaggressions and W3 (i.e., persons with a disability/Indigenous vs. racialized members) was also marginally significant, *t* = -1.6771, *p* = .0936, such that microaggressions were a stronger predictor for racialized members than for PwD and Indigenous members.

### Organizational inclusion and intentions to leave

The interaction between organizational inclusion and designated group membership was not significant, Wald *F*(4, 3967) < 1.

1. Analyses were also conducted by excluding gender diverse individuals from the "Everyone else" group (13 survey respondents), and all results remained unchanged. Results including gender diverse individuals are reported herein, for greater inclusivity and sample size. [↑](#footnote-ref-1)
